# Supplementary material for: Inhibition of LncRNA Kcnq1ot1 suppresses hypoxia-induced pyroptosis of H9C2 cells by regulating miR-27b-3p
Source: PLoS One. 2025 Sep 18;20(9):e0332892. doi: 10.1371/journal.pone.0332892 (PMC12445483; doi:10.1371/journal.pone.0332892)

Original image for Figure 1B

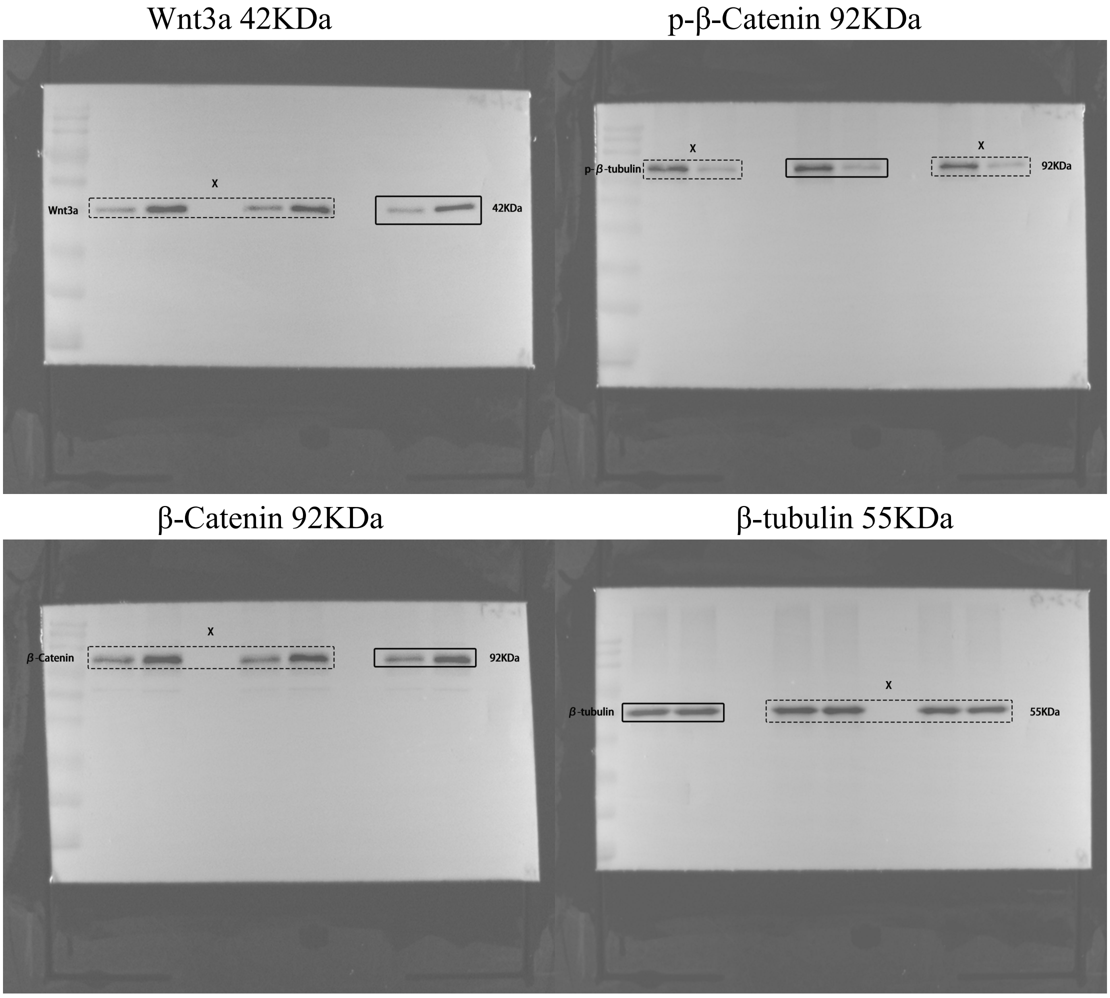

Original image for Figure 1D

NLRP3 110KDa

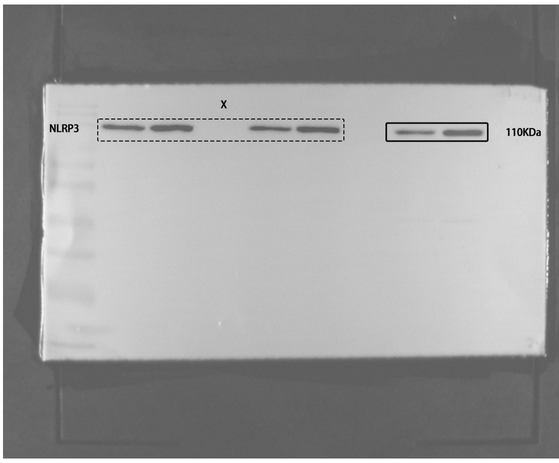

Caspase-1 40KDa

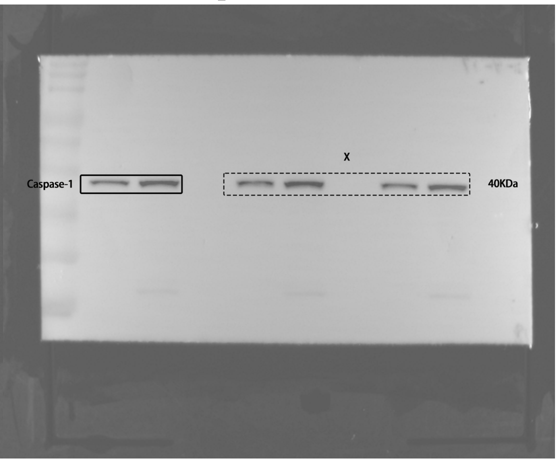

β-tubulin 55 KDa

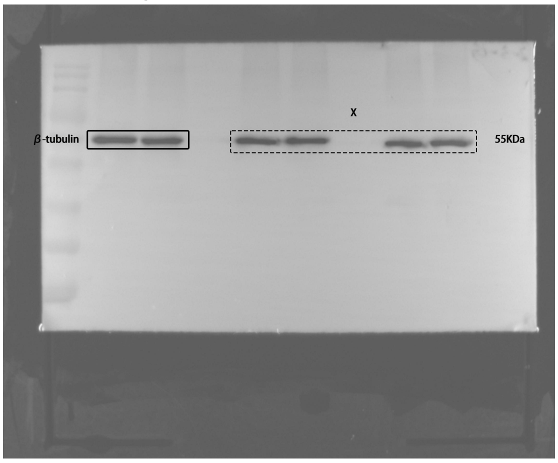

Original image for Figure 1G

Fibronectin 263KDa

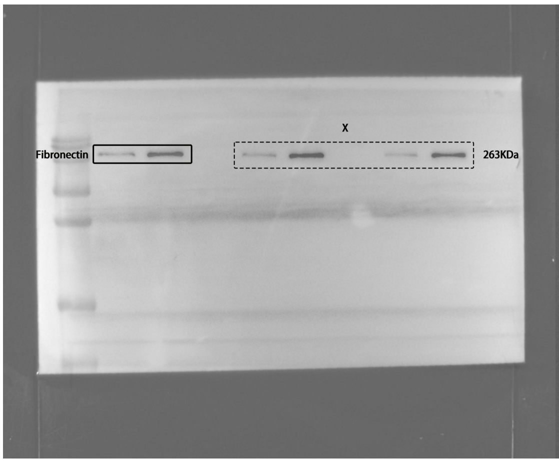

Collagen3 150KDa

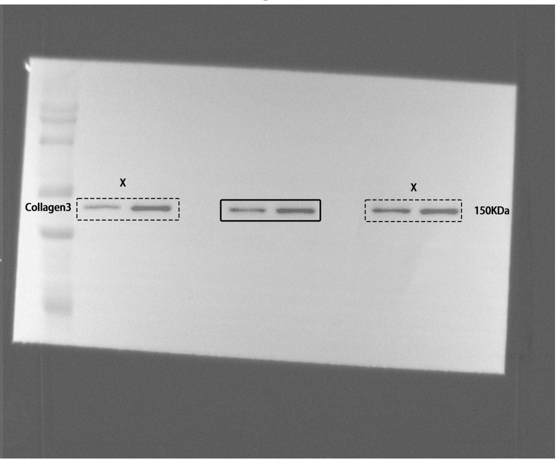

β-tubulin 55KDa

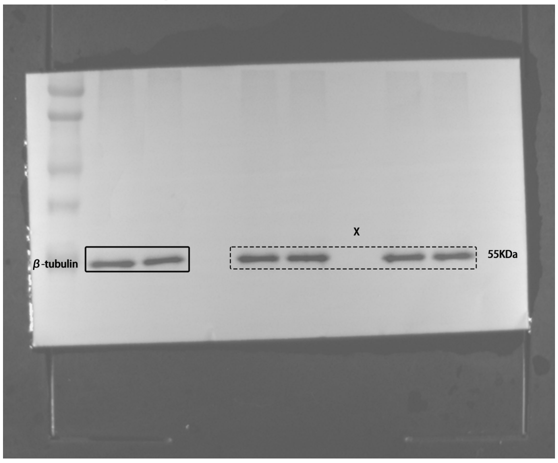

Original image for Figure 2D

Wnt3a 42KDa

p-β-Catenin 92KDa

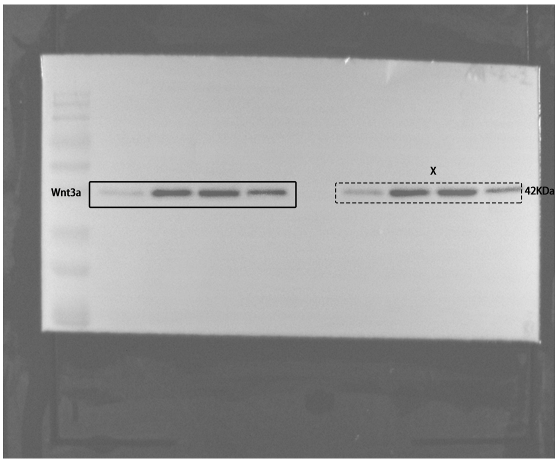

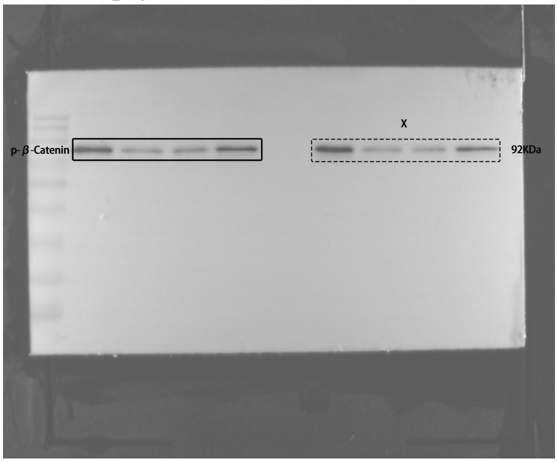

β-Catenin 92KDa

β-tubulin 55KDa

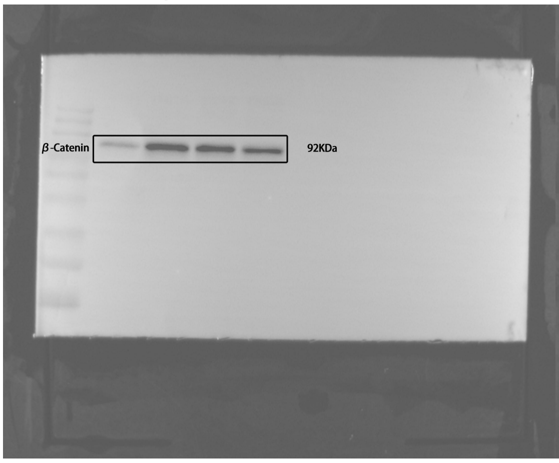

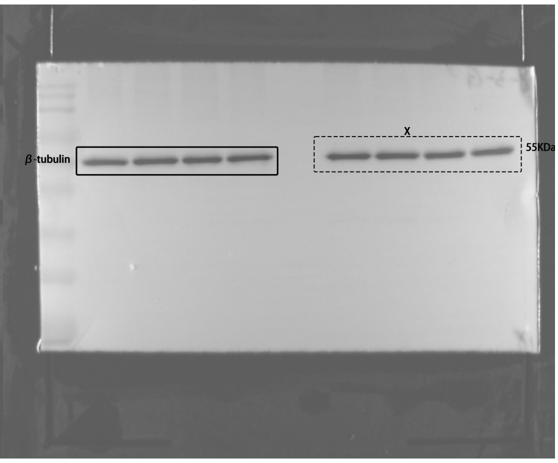

Original image for Figure 2F

NLRP3 110KDa

Caspase-1 40KDa

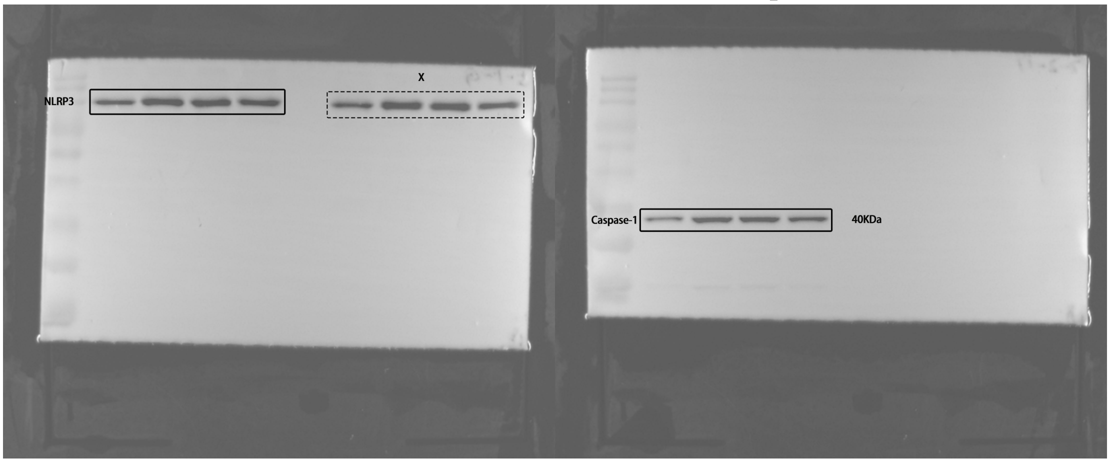

β-tubulin 55KDa

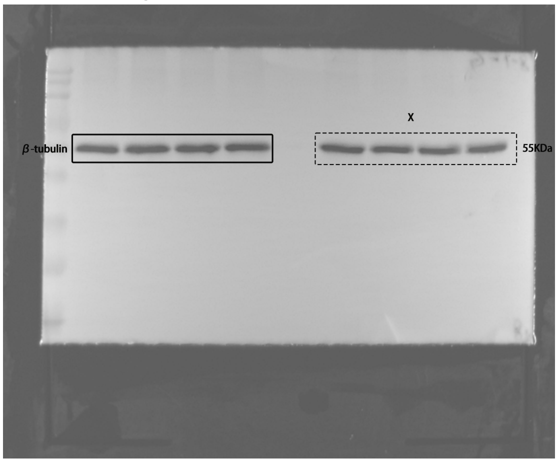

Original image for Figure 2I

Fibronectin 263KDa   Collagen3 150KDa

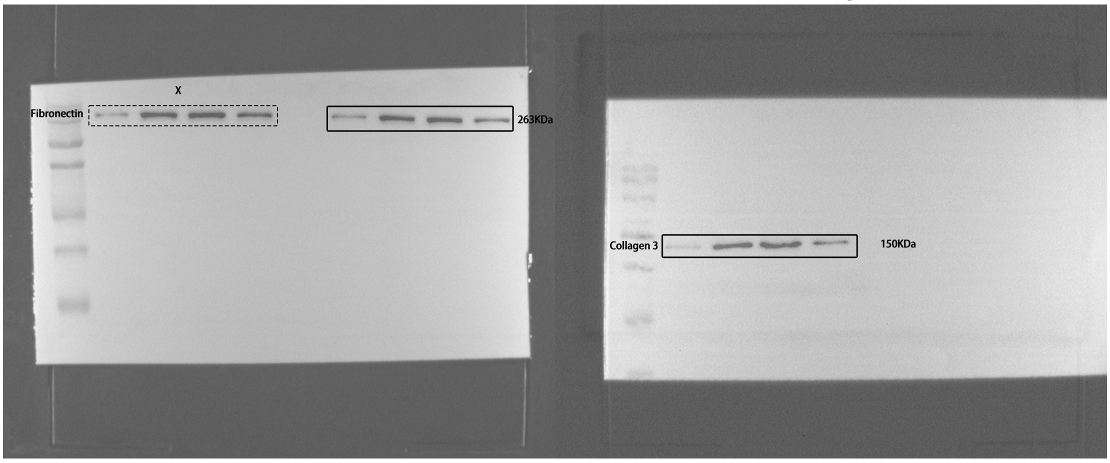

β-tubulin 55KDa

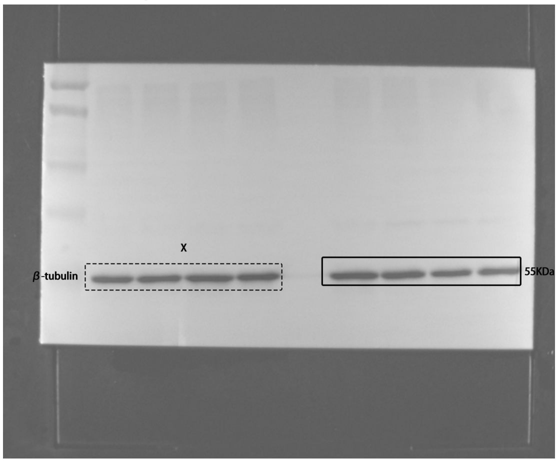

Original image for Figure 3D

Wnt3a 42KDa

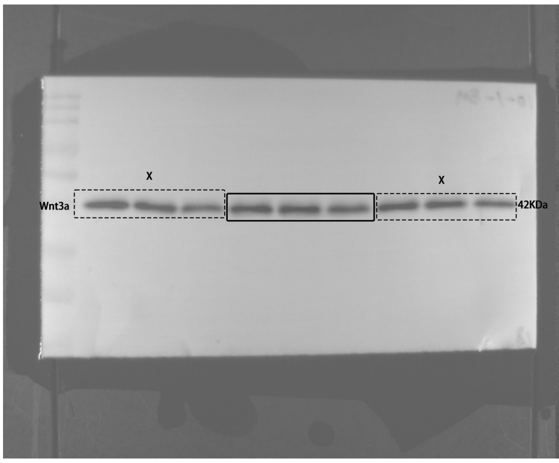

p-β-Catenin 92KDa

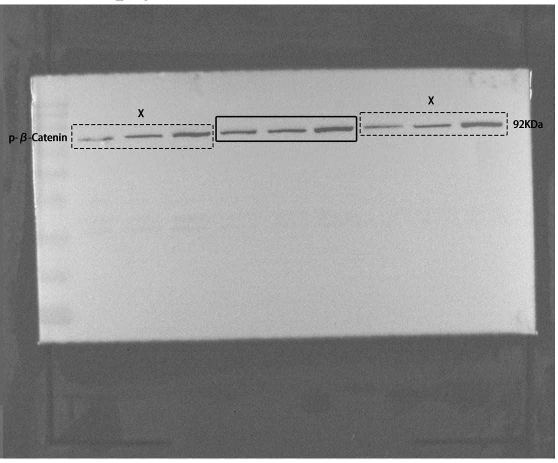

β-Catenin 92KDa

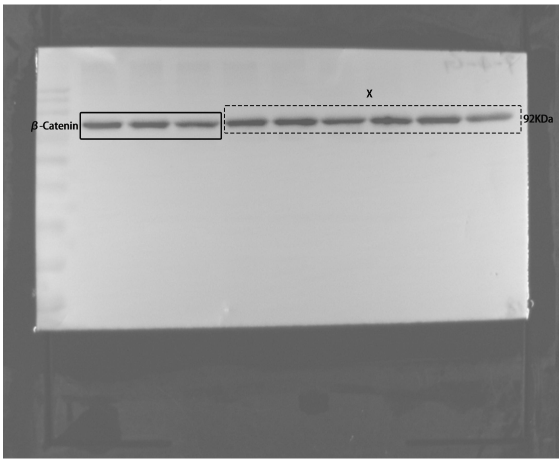

β-tubulin 55KDa

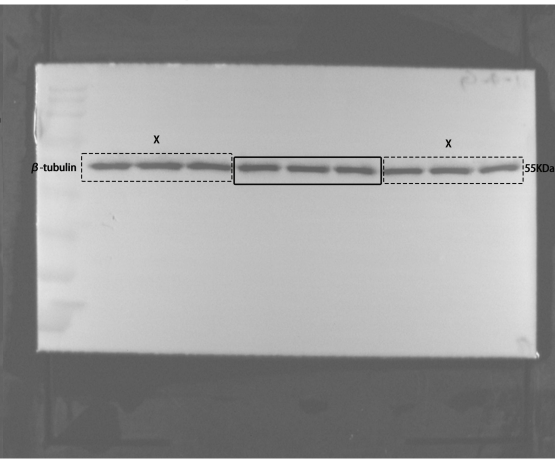

Original image for Figure 3F

NLRP3 110KDa

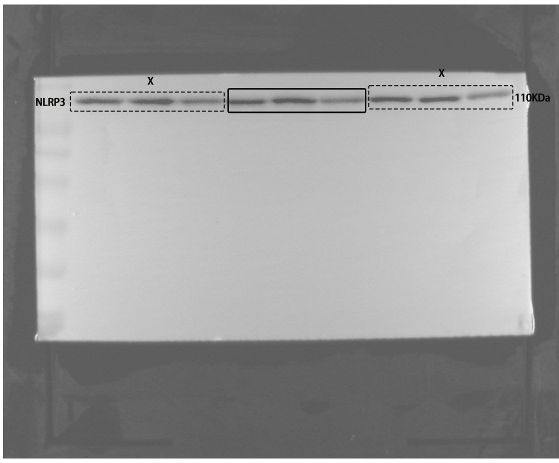

Caspase-1 40KDa

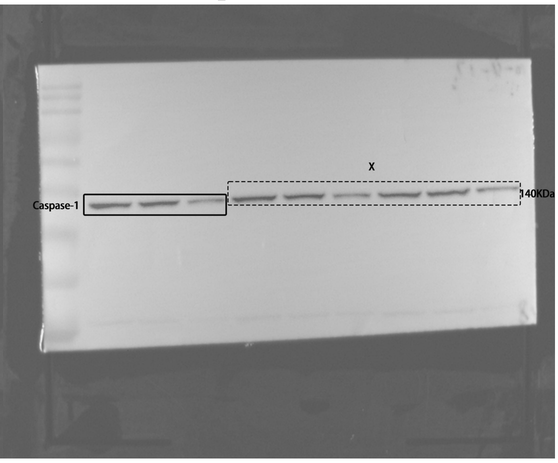

β-tubulin 55KDa

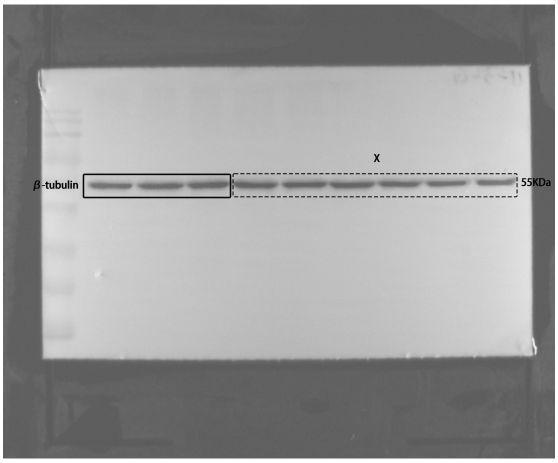

Original image for Figure 3I

Fibronectin 263KDa

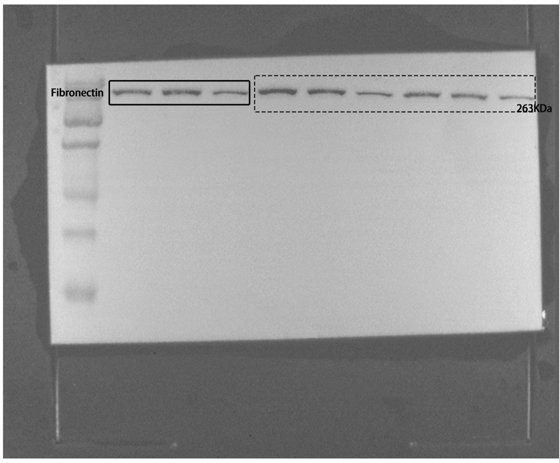

Collagen3 150KDa

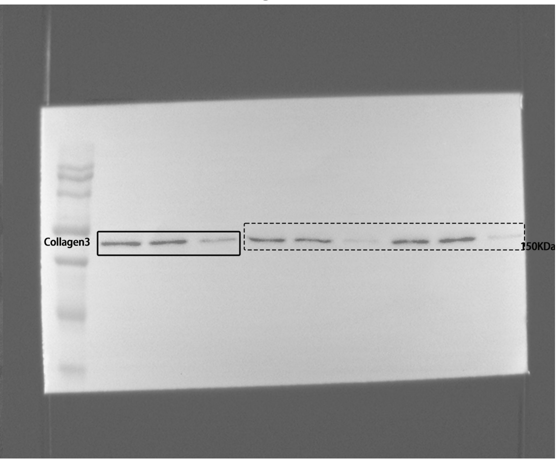

β-tubulin 55KDa

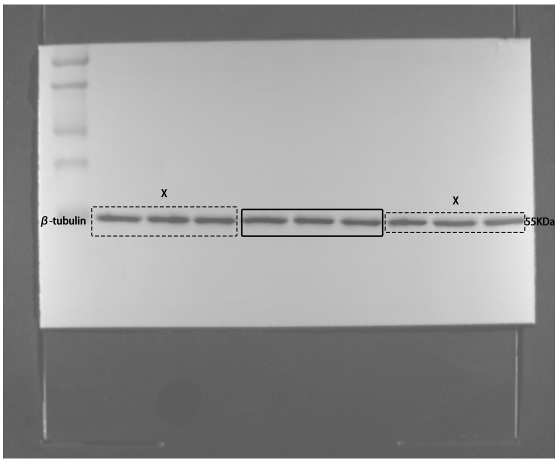

Original image for Figure 5C

Wnt3a 42KDa

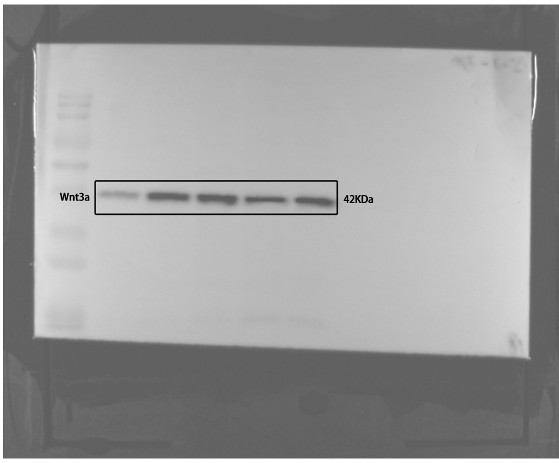

p-β-Catenin 92KDa

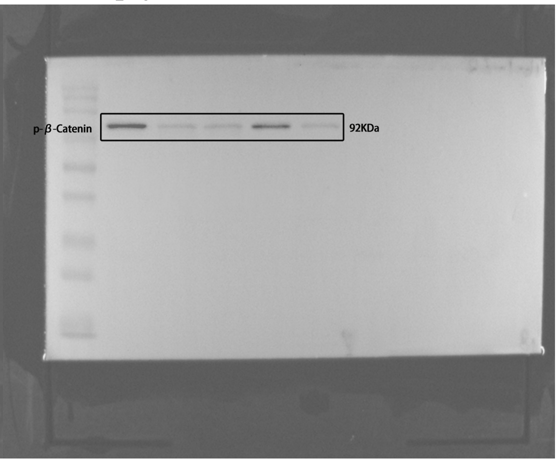

β-Catenin 92KDa

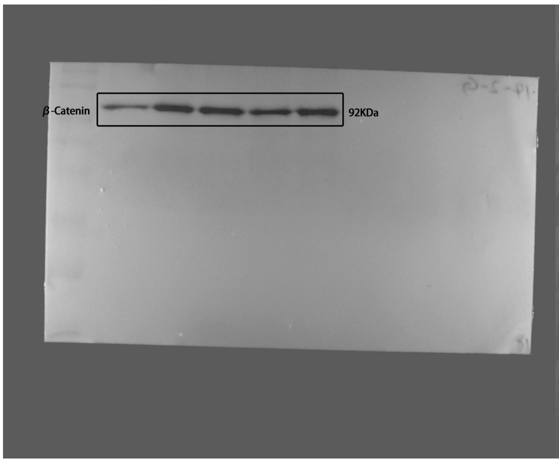

β-tubulin 55KDa

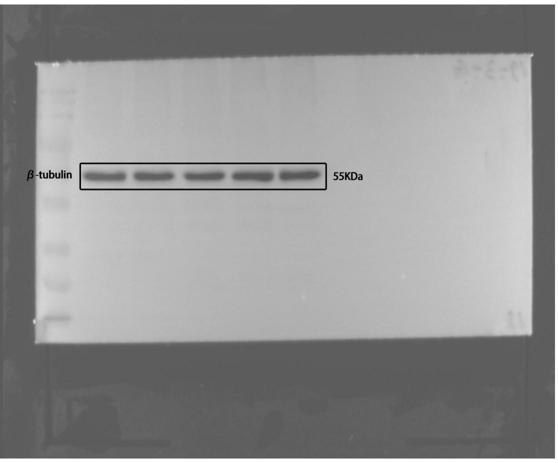

Original image for Figure 5E

NLRP3 110KDa

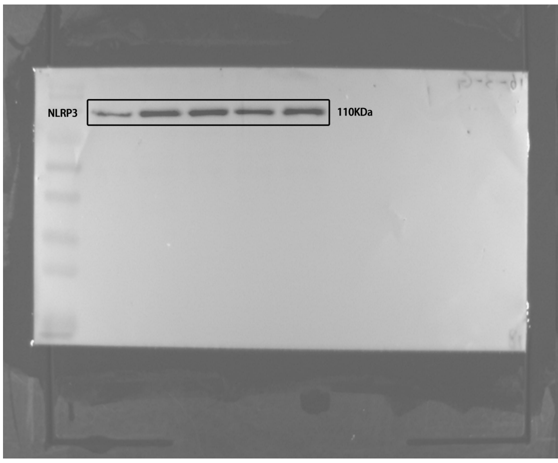

Caspase-1 40KDa

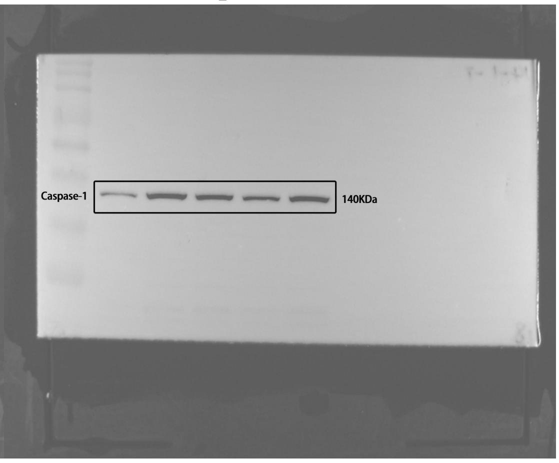

β-tubulin 55KDa

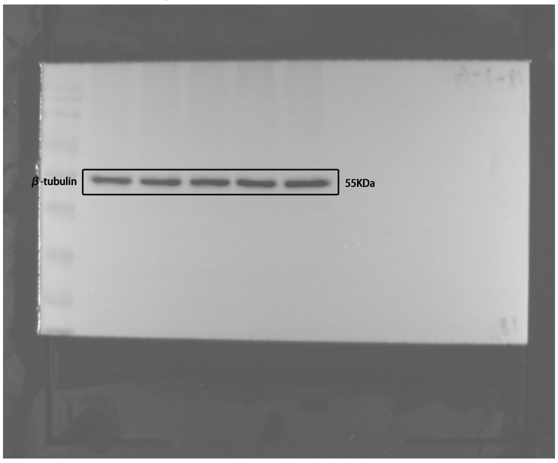

Original image for Figure 5H

Fibronectin 263KDa

Collagen3 150KDa

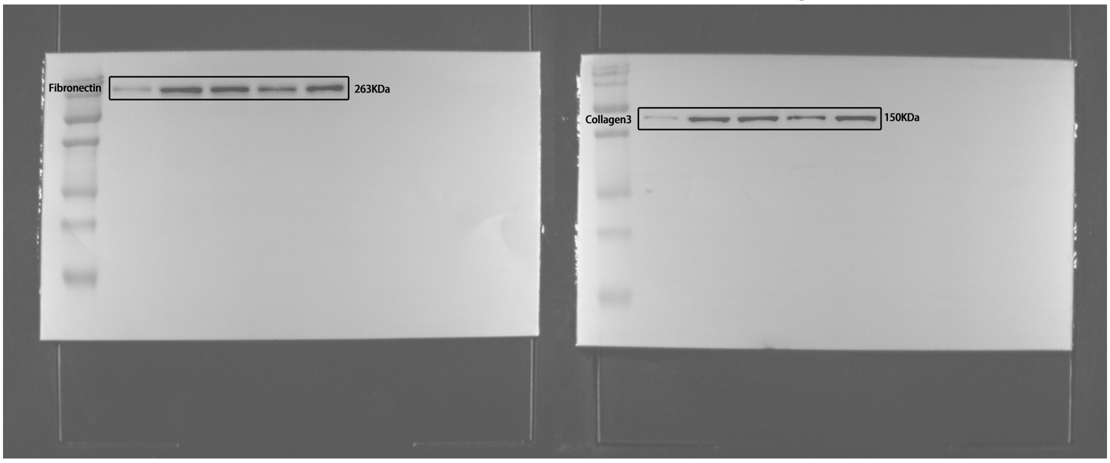

β-tubulin 55KDa

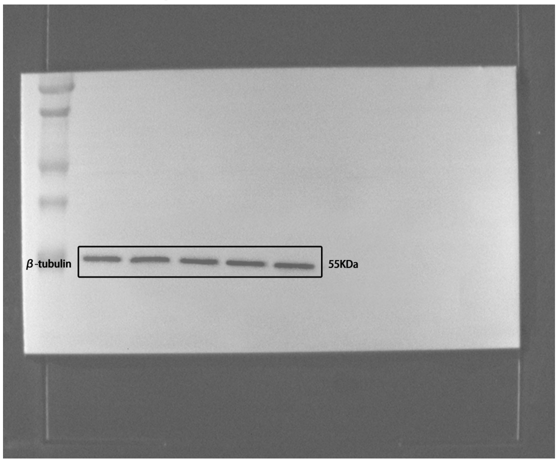

Supplement: S1 File — (PDF) [file pone.0332892.s001.pdf]
